# Prescription Pattern of Antidepressants and the Potential for Personalized Medicine in the Qatari Population

**DOI:** 10.3390/jpm11050406

**Published:** 2021-05-13

**Authors:** Kholoud Bastaki, Mohammed El Anbari, Suhaila Ghuloum, Puthen Veettil Jithesh

**Affiliations:** 1 College of Health & Life Sciences, Hamad Bin Khalifa University, Doha 34110, Qatar; khbastaki@hbku.edu.qa; 2 Sidra Medicine, Doha 26999, Qatar; melanbari@sidra.org; 3 Mental Health Services, Hamad Medical Corporation, Doha 3050, Qatar; sghuloum@hamad.qa; 4 Weill Cornell Medicine, Doha 24144, Qatar

**Keywords:** antidepressants, prescription pattern, Middle East

## Abstract

Studying the prescription pattern of medications will help in understanding potential unnecessary prescriptions, due to the trial-and-error method of prescribing, and the need for personalized medicine in a population. Therefore, in this study, our aim was to explore the prescribing pattern and off-label use of antidepressants in the Qatari population. We conducted a retrospective study of Qatari patients who received prescriptions for antidepressants from the major healthcare providers in Qatar, for a period of 24 months between June 2018 and May 2020. The number of patients, prescriptions, and diagnostic indications were analyzed. The chi-square test was used for identifying statistically significant association of the number of individuals prescribed with age category or gender. Of the 14,601 Qatari patients who were prescribed antidepressants, the majority were female (61%, *p* < 2.2 × 10^−16^), and were at or above 60 years of age (27%, *p* < 2.2 × 10^−16^). More numbers of selective serotonin reuptake inhibitors (SSRIs) (22,085 out of 48,031; 46%), were dispensed than other classes of antidepressants, with escitalopram (26%) at the top of the list. Preponderance of prescription of antidepressants for non-mental health diseases was observed. Population-level prescription trends, as we reported here, when combined with patient genetic variability and outcome data, will have the power to predict the potential for treatment failures and adverse effects of these medications in the population. We also recommend educating non-mental health prescribers about the adherence to evidence and guidelines to ensure patient safety while prescribing antidepressants.

## 1. Introduction

The burden of mental illness is increasing across the world; more than one in ten are estimated to live with a mental health disorder (MHD) [1]. In the eastern Mediterranean region, Qatar ranked second for premature mortality from MHDs [2]. In Qatar, almost 37% of adults receiving healthcare from a primary healthcare setting met diagnostic criteria for at least one MHD, with depression being the most commonly diagnosed MHD (14%), followed by anxiety disorders (10.3%) [3]. Another study from Qatar identified that 20% of participants had a generalized anxiety disorder, while 19% had a depressive disorder, and both studies showed that women had a higher risk of MHD [4].

An increase in the usage of antidepressants over the years was observed. For example, the introduction of selective serotonin reuptake inhibitors (SSRIs) in the late 1980s resulted in increased prescriptions of antidepressants, which doubled in 10 years in western countries [5]. An increase in length of the prescribing period was also observed, putting patients at higher risk of adverse effects; this often leads to an increased rate of mortality, especially in elderly patients [6]. The use (and overuse) of antidepressants for mental illnesses are often debated (i.e., people are either for or against it) [7,8,9]. However, their use in treating non-mental health diseases have also become widespread, even without guidelines or evidence for their appropriateness in such conditions [10,11,12]. Thus, understanding the prescription rates and patterns of antidepressants is important to improve the personal and economic burdens associated with unnecessary use of medications.

It is a major concern that some patients may not respond as effectively to medications, but have adverse effects. In different types of MHDs, only 30% of patients reach full remission, and continue their medications [13,14,15,16]. This has raised a call for better use of antidepressants, with emphasis on searching for predictors of responses, and reducing the usage in people with situational and personality-based problems. Drug response varies between individuals due to several factors, including disease heterogeneity, and genetic and environmental factors [13]. Evidence indicates that genetic factors play an important role in determining the differences in response to antidepressants and adverse effects [15,17,18].

Our recent investigation of the pharmacogenomic profile of an apparently healthy adult Qatari population, from whole genome sequencing data, identified that a large proportion carry variants in genes, *CYP2C19* and *CYP2D6*, which are important in the metabolism of several antidepressants [19]. Based on the guidelines available from the Clinical Pharmacogenetic Implementation Consortium (CPIC), phenotypic effects of these variations (metabolizer status) can predict higher risk of non-response or side effects due to the use of these drugs [20,21]. However, data on the prescriptions of these medications in the Qatari population are limited—data that are important in making informed decisions, i.e., on the need to implement pharmacogenomic testing in the country [22]. Therefore, in this study, our aim was to explore the prescribing patterns and off-label use of antidepressants in the Qatari population.

## 2. Materials and Methods

### 2.1. Patient Population

We conducted a retrospective database study of Qatari patients who received prescriptions for antidepressants from the major healthcare providers in Qatar, Hamad Medical Corporation (HMC), and Primary Healthcare Corporation (PHCC), for a period of 24 months between June 2018 and May 2020. The inclusion criteria for the study were (a) Qatari patients who were visiting outpatient clinics at HMC and PHCC between June 2018 and May 2020, and (b) prescribed antidepressants during the study period. Exclusion criteria included (a) non-Qatari patients, and (b) patients who were hospitalized (inpatients). Data points collected and the medications targeted are provided in the Supplementary Information. Only anonymized datasets were analyzed after obtaining an approval waiver from the HMC. Electronic records of prescription data from a cohort of 14,601 patients who received antidepressant prescriptions and 9349 patients who were prescribed antipsychotics were mined. We calculated the number of patients who were prescribed various antidepressants and antipsychotics as well as the actual number of prescriptions dispensed.

Anatomical Therapeutic Chemical classification (ATC) codes associated with the drug categories were obtained from the database of WHO Collaborating Centre for Drug Statistics Methodology [23] and CPIC levels of drug-gene pairs were obtained from the Clinical Pharmacogenetics Implementation Consortium database [24].

### 2.2. Statistical Analysis

We performed the chi-square test for the association between the drugs and the age category, and for the association between the drugs and gender. Prescription counts were summarized using balloon graphics, where the size of each balloon represented the magnitude of the count at a given point, while crossing the two variables. R statistical package was used for the analysis and plotting.

## 3. Results

### 3.1. Prescription Pattern of Antidepressants

**Figure 1:**
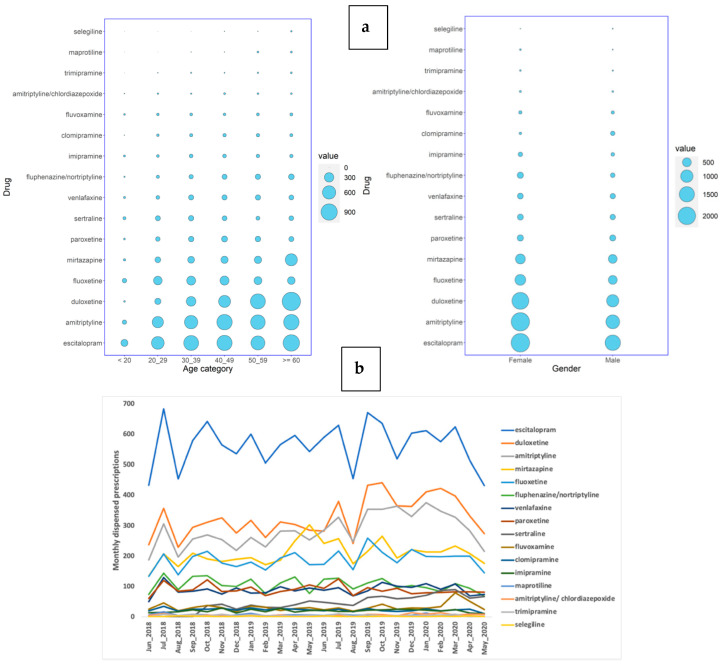
(**a**) The distribution of the number of Qatari patients receiving prescriptions of antidepressants over a period of 2 years in all the HMC/PHCC hospitals stratified by age or gender, and (**b**) the number of prescriptions of antidepressants dispensed over a period of 2 years in the HMC/PHCC hospitals, with the number of prescriptions dispensed monthly on the *Y*-axis and the months on the *X*-axis.

Of the 14,601 Qatari patients who were prescribed antidepressants at the major healthcare providers during the 24 months between June 2018 and May 2020, the majority were female (8847; 61%), and the gender difference was statistically significant (chi-square *p* < 2.2 × 10^−16^). The majority of the antidepressant prescriptions were given to patients at or above 60 years of age (4004; 27%), showing statistically significant differences among the age groups (chi-square *p* < 2.2 × 10^−16^) (Figure 1a; Supplementary Table S1a). Six non-selective monoamine reuptake inhibitors were prescribed for the Qatari population, including one tetracyclic antidepressant (maprotiline) and five tricyclic antidepressants (TCAs; amitriptyline alone and in combination with chlordiazepoxide, nortriptyline with fluphenazine, clomipramine, imipramine, and trimipramine). Five selective serotonin reuptake inhibitors (SSRIs) were also prescribed, escitalopram, fluoxetine, paroxetine, sertraline, and fluvoxamine. Other antidepressants prescribed included duloxetine, mirtazapine, and venlafaxine. Escitalopram (3775; 26%) was the most highly prescribed antidepressant for these patients, followed by amitriptyline (3400; 23%) and duloxetine (2816; 19%) (Figure 1a).

There were 48,031 antidepressant prescriptions dispensed for Qataris over the 24 months. SSRIs were the most highly prescribed (22,085; 46%), followed by ‘other antidepressants’ (ATC: N06AX) (15,033; 31%), and tricyclic antidepressants (10,689; 22%). Specifically, escitalopram (13,587; 28%), was the most commonly dispensed, followed by duloxetine (7871; 16%) and amitriptyline (6825; 14%) (Figure 1b).

### 3.2. Diagnostic Indications for Antidepressant Prescriptions

**Figure 2:**
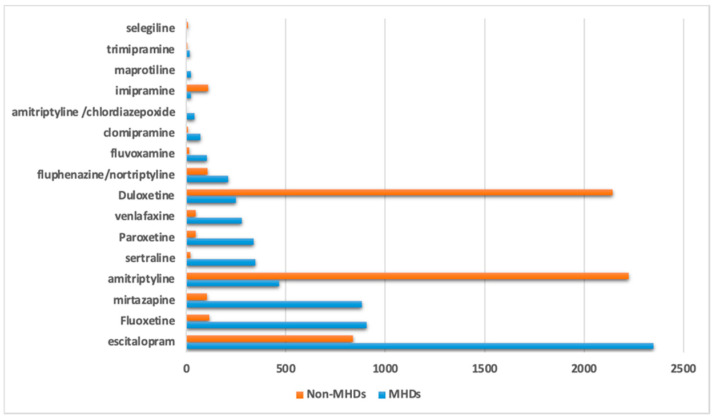
Diagnostic indications for antidepressant prescriptions in the Qatari population for mental health disorders (MHDs) and non-MHDs.

There were 8386 diagnostic indications recorded, which may be classified into mental health disorders (MHDs), and 6503 as non-MHDs. The prescription of duloxetine, amitriptyline, and imipramine were especially more for non-MHDs compared to MHDs, while several other antidepressants, including escitalopram, were also prescribed for non-MHDs (Figure 2).

**Figure 3:**
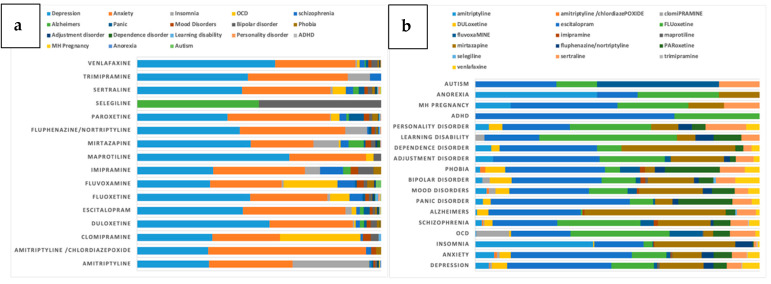
Use of antidepressants for the treatment of mental health disorders in the Qatari population, stratified into various categories and displayed based on (**a**) drugs, and (**b**) diagnoses. OCD: Obsessive compulsive disorder, ADHD: Attention-deficit hyperactivity disorder.

Major diagnostic indications for MHDs included depression (3655; 44%) and anxiety (3039; 36%), followed by insomnia (444; 5%), obsessive compulsive disorder (OCD, 269; 3%) and schizophrenia (215; 3%) (Figure 3a). Escitalopram was the main antidepressant prescribed for depression (1338; 37%), followed by mirtazapine (566; 16%) and fluoxetine (549; 15%). For anxiety, escitalopram was the major prescription (1293; 43%), followed by fluoxetine (372; 12%) and mirtazapine (311; 10%) (Figure 3b).

**Figure 4:**
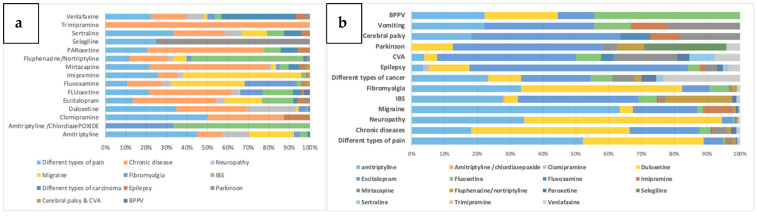
Non-mental health disease (non-MHD) use of antidepressants in the Qatari population, stratified into various non-MHD categories and displayed based on the (**a**) drugs, and (**b**) diagnoses. BPPV: benign paroxysmal positional vertigo, CVA: Cerebrovascular accident, IBS: Irritable bowel syndrome.

Non-mental health diagnoses observed for antidepressants included different types of chronic pain (2050; 32%), chronic diseases (e.g., cardiovascular diseases, hypertension, diabetes mellitus etc.; 1753; 27%), neuropathy (965; 15%), migraine (965; 15%), irritable bowel syndrome (IBS; 340; 5%), and fibromyalgia (204; 3%), among others (Figure 4a). Amitriptyline (1069; 52%) followed by duloxetine (749; 37%) were mainly prescribed for chronic pain, while duloxetine was highly prescribed for chronic diseases (844; 48%) and neuropathy (581; 60%). Escitalopram (374; 21%) and amitriptyline (316; 18%) were also prescribed highly for chronic diseases, while amitriptyline (331; 34%) was also highly prescribed for neuropathic pain. Another major use of amitriptyline was for migraine (612; 24% of its total use for non-MHDs) (Figure 4b).

For the on-label and off-label use of antidepressants, we found that 48% of prescriptions were following the United States Food and Drug Administration (FDA) approved indications while the majority of the indications of antidepressants (52%) were for FDA unlicensed use, such as for controlling chronic diseases, sleeping disorders, and schizophrenia.

### 3.3. Prescription Pattern of Antidepressants in the Mental Health Hospital

**Figure 5:**
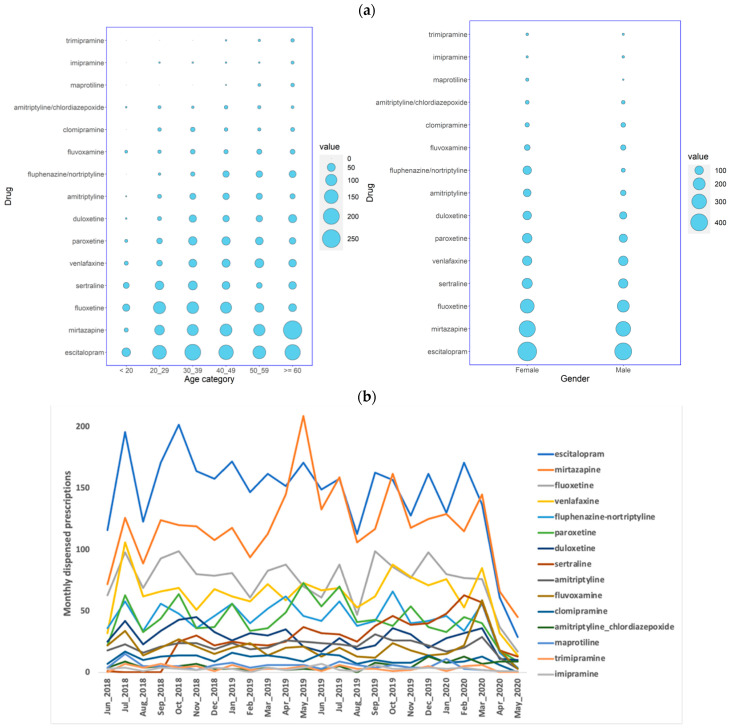
(**a**) The distribution of the number of Qatari patients receiving prescriptions of antidepressants over a period of 2 years in the Mental Health Hospital alone, stratified by age or gender, and (**b**) the number of prescriptions of antidepressants dispensed over a period of 2 years in the Mental Health Hospital alone, with the number of prescriptions dispensed monthly on the *Y*-axis, and the months on the *X*-axis.

As the prescription of antidepressants were high for non-MHD indications in the general hospital setting, we also studied the prescription pattern specifically for patients attending the Mental Health Hospital (MHH). Of the 3395 patients, there were more women than men who were prescribed antidepressants at MHH during the 24 month period (1915; 56%; chi-square *p* = 3.45 × 10^−6^). Age category also showed statistically significant association with the number of patients prescribed (chi-square *p* < 2.2 × 10^−16^), with those ≥60 years receiving the greatest number of prescriptions (796; 23%) (Figure 5a; Supplementary Table S1b). When the MHH alone was considered, among the 14,771 prescriptions dispensed, escitalopram (3492; 24%) was still the most highly prescribed antidepressant, but was followed by mirtazapine (2857; 19%) and fluoxetine (1807; 12%) (Figure 5b). The most common diagnoses reported in the MHH were depression and anxiety disorders.

## 4. Discussion

In our study of the prescription patterns in the Qatari population, we found that antidepressants were prescribed more for women and ages at or above 60 years. A Swedish study also reported that prescribing of antidepressants was high in women and aged 45–64 years [25]. Indeed, MHDs are a concern in older adults and it affects around 15% of individuals who are 60 years or older, with an increased risk of mortality and morbidity [26]. However, the use of antidepressants in such groups should be closely monitored due to safety issues and adverse effects. Several studies about prescribing patterns of antidepressants showed that the majority of the patients receiving these medications were females [25,27]. Depression and anxiety disorders occur more frequently in women than in men and is usually 1.5–3 times higher [28]. Moreover, many global reports showed that depression is the leading cause of disease-related disability in women. Sometimes mood changes in women happen with normal hormonal changes, such as pre-menopause, menstrual cycle, during pregnancy, and postpartum, which makes them more susceptible to develop anxiety and depression. However, hormonal changes may not be the only reason; these gender differences are probably due to many factors, including biological, inherited traits, demographic, psychological, or social effects. Gender itself affects many aspects of psychopathology of MHDs, such as the onset of symptoms, prevalence of these disorders, treatment plan, medication selection (e.g., some medications are not given to pregnant woman or those planning to get pregnant) and treatment response, and even in their help-seeking behavior [29]. Although, women experience depressive episodes and anxiety disorders more often than men, men appear to have early onset of serious MHDs, such as schizophrenia and bipolar disorder than women [28,30,31]. The onset of bipolar disorder tends to occur later in women. Moreover, substance use disorders are more common in men [32].

SSRIs were the major class of antidepressants prescribed in our cohort. This finding is in parallel with studies from other countries [33]. However, escitalopram was the most prescribed antidepressant for the Qatari population, while in some other countries, sertraline was the most commonly prescribed, followed by escitalopram or fluoxetine [34]. The reason for the higher level of prescription of escitalopram compared to other SSRIs may be manifold. Such contributing factors include better efficacy and tolerability of escitalopram, or even the availability. For example, sertraline was introduced in the hospitals we studied only recently, and its prescribing privileges are restricted to specialized physicians. On the other hand, escitalopram was available to HMC and PHCC hospitals with wider prescribing privileges. Even though SSRIs have better safety and tolerability profiles than some of the older antidepressants, long-term use can lead to significant side effects, including sleep disturbances, weight gain, and sexual dysfunction, among others [35]. Increasing risk of adverse effects and mortality due to antidepressant use was also observed along with increasing age, with patients aged 65 and above more seriously affected. Thus, the trend of antidepressant prescriptions observed in the Qatari population may point towards the need for careful monitoring of side effects, especially in elderly patients, and finding alternate solutions.

Some interesting patterns were also observed in the prescription numbers. There were distinctive dips in the month of August in both years, coinciding with the holiday season, characterized by the large outflow of residents due to the extreme hot temperatures in the country. A distinct dip was also observed in May 2020, especially in the number of prescriptions from the MHH, due to closure of clinics following the COVID-19 outbreak.

For antidepressants, when FDA approved indications (on-label and off-label use) were considered, our study found variations in the most commonly prescribed medications between the Mental Health Hospital (MHH) and different facilities at the general hospitals in the HMC and PHCC. The differences are due to the use of some antidepressants, especially TCAs, for indications such as migraine, neuropathic pain, and different types of pain management. Both TCAs and serotonin-norepinephrine reuptake inhibitors (SNRIs) possess analgesic qualities, while the evidence for the effectiveness of SSRIs is weaker. Several studies have shown that antidepressants were effective in relieving neuropathic pain. Antidepressants might also provide effective pain relief for other painful conditions, such as chronic musculoskeletal pain and fibromyalgia. Since the pain relief effects of antidepressants seem to occur earlier, and at lower doses than for antidepressant effects, it was suggested that the mechanism of action to control the pain is separate from their antidepressant effects. However, none of the TCAs was approved by the FDA for any pain management indications since their mechanisms of action are unclear.

Duloxetine is the only antidepressant that has FDA approval for the treatment of neuropathic pain and chronic musculoskeletal pain. In addition, duloxetine and milnacipran were approved for fibromyalgia. Depression and chronic pain are conditions having some overlap in their symptoms. A positive association between depression and chronic pain was also observed. An analysis of data from the National Health and Nutrition Examination showed that 90% of patients with depression were also having pain symptoms [36]. The link between these two conditions could have biological basis besides the psychological effect. For example, neurotransmitters, such as serotonin and norepinephrine, were implicated in the underlying pathophysiology of depression and chronic pain. Therefore, recent guidelines from the UK National Institute for Health and Care Excellence (NICE) recommends the use of TCAs, SNRIs, and some anticonvulsants acting at calcium channels, such as pregabalin and gabapentin as first-line medications for the treatment of patients with neuropathic pain [37].

In Qataris, amitriptyline was the most highly prescribed antidepressant for different types of pain, followed by duloxetine and escitalopram. In the case of neuropathy, 60% of the patients were prescribed duloxetine followed by amitriptyline (34%). An off-label use of amitriptyline was for treating migraine, followed by escitalopram. According to the American Academy of Neurology and the American Headache Society guidelines, amitriptyline was considered for migraine prophylaxis, the only antidepressant with consistent evidence supporting its effectiveness for this use. In addition, studies found that amitriptyline was more beneficial for patients with mixed migraine and tension features [38].

Antidepressants were also used in Qatar to treat some patients with irritable bowel syndrome (IBS). Meta-analyses of cumulative controlled experience have confirmed the efficacy of antidepressants in IBS and other functional gastrointestinal disorders [39]. According to the National Institute for Health and Care Excellence (NICE) guideline, antidepressants, particularly TCAs, were considered as second-line treatment for people with IBS, if other medications, such as laxatives or antispasmodics, did not help. Moreover, SSRIs should be considered for patients with IBS only if TCAs are ineffective [40].

Another common off-label use highlighted in our study was the use of antidepressants, especially amitriptyline and mirtazapine, for sleeping disorders. The use of antidepressants for treating sleeping disorders, particularly insomnia, is widespread. Nevertheless, none of the antidepressants is licensed for treating sleeping disorders, except doxepin, which is the only TCA that was approved by the FDA for the treatment of insomnia. This type of use for antidepressant medications may be driven by concern over longer-term use of hypnotics and the limited availability of psychological treatments for insomnia [41].

Interindividual variability in response to antidepressants can affect the treatment outcome and lead to potential adverse effects. Pharmacogenomics testing of variants known to be associated with such variability can help in the personalized prescription or dosage adjustments for increased efficacy and reduced toxicity [42]. Consortia, including the Clinical Pharmacogenetic Implementation Consortium (CPIC) and the Dutch Pharmacogenetic Working Group (DPWG), issue guidelines that help in the clinical implementation of pharmacogenomic testing. However, clinical implementation of pharmacogenomics was slow worldwide, especially in the Middle East, including Qatar, due to the lack of population-specific genetics, prescriptions, and outcome data [22]. Currently five antidepressants have the highest level of evidence and guidelines by the CPIC (Level A) [24], of which four were prescribed in Qatar (amitriptyline, nortriptyline, escitalopram, and paroxetine).

Furthermore, our recent investigation of the pharmacogenomic landscape of the Qatari population identified the high prevalence of actionable variation in the genes, *CYP2C19* and *CYP2D6* [19]. Both these genes code for cytochrome P450 enzymes critical in the metabolism of antidepressants, such as SSRIs and TCAs, and there are guidelines available for the clinical implementation of pharmacogenomic tests for identifying variation in these genes and how these test results can be interpreted for adjusting the dosage or suggesting alternate drugs [20,21]. Since escitalopram and amitriptyline are among the major antidepressants prescribed to Qatari patients, our results highlight the importance of implementation of pharmacogenomic testing prior to prescribing these medications, in order to prevent unnecessary hospitalizations and inefficient treatment regimens. This will in turn reduce the associated economic burden and improve the quality of life of the patients. Pharmacogenomic testing will be particularly useful when these drugs are used in the treatment of mental health conditions as larger doses are administered compared to their use in non-mental health conditions.

It is also worth noting that, with the emergence and proliferation of direct-to-consumer genomic testing in some parts of the world, more patients are testing for genetic markers, for response to psychotropic medication, at a very high cost, with (currently) little clinical impact. In particular, the younger generation, who are more aware, often ask for such tests or enquire about them, preferring to have a confirmed/evidence-based personalized medication selection rather than trial and error.

## 5. Conclusions

With the accumulation of big datasets generated from multiple technologies, and advancements in artificial intelligence and machine learning methodologies, the field of psychiatry is also progressing towards precision and personalized medicine approaches [43]. Population-level prescription trends, as we reported here, when combined with patient genetic variability and outcome data from these treatment modalities, will have the power to predict the potential for treatment failures and adverse effects of these medications in the population. Such information will help influence policy makers to understand utilization and form evidence-based guidelines for better treatment options. We also recommend educating non-mental health prescribers on prescribing antidepressants without enough evidence, to ensure that appropriate tests are undertaken when necessary, side effects are monitored, and patient safety is maintained.

## Data Availability

The data are either presented in full or available through the HMC Abhath portal (https://abhath.hamad.qa/abhath, (last accessed on 5 March 2021)) after approval following registration and application.

## Supplementary Materials

The following are available online at https://www.mdpi.com/article/10.3390/jpm11050406/s1, Table S1: The number of Qatari patients receiving prescriptions of antidepressants over a period of 2 years in (a) all the hospitals, and (b) mental health hospital alone.
