# Supplementary material for: Prescription Pattern of Antidepressants and the Potential for Personalized Medicine in the Qatari Population
Source: J Pers Med. 2021 May 13;11(5):406. doi: 10.3390/jpm11050406 (PMC8152751; doi:10.3390/jpm11050406)
Supplement: Supplementary file 1 [file jpm-11-00406-s001.zip › jpm-1180874-supplementary.pdf]

Journal of Personalized Medicine

MDPI

Supplementary Information:

Variables collected:

Nationality, age and gender, date of prescription, organization, name of medications, dose of medications, quantity dispensed, diagnosis related to prescribed antidepressants.

List of medications:

1. Amitriptyline 10 mg TAB
2. Amitriptyline 25 mg TAB
3. Amitriptyline/Chlordiazepoxide 12.5 mg/5 mg TAB
4. clomiPRAMINE 25 mg TAB
5. clomiPRAMINE 75 mg (sustained release) TAB
6. DULoxetine 30mg CAP
7. DULoxetine 60mg CAP
8. Escitalopram 10 mg TAB
9. Escitalopram 20 mg TAB
10. Fluoxetine 20mg CAP
11. fluPHENAZine HCl/Nortriptyline HCl 500 mcg/10 mg TAB
12. Fluvoxamine 100 mg TAB
13. fluvoxaMINE 50 mg TAB
14. Imipramine 10 mg TAB
15. Imipramine 25 mg TAB
16. Maprotiline 25 mg TAB
17. Maprotiline 50 mg TAB
18. Mirtazapine 30 mg TAB
19. PARoxetine 20 mg TAB
21. Selegiline 5 mg TAB
22. Sertraline 50mg TAB
26. Trimipramine 25 mg TAB
27. Trimipramine 50 mg TAB
28. Trimipramine 50mg CAP
29. Venlafaxine 150mg (sustained release) CAP
30. Venlafaxine 37.5mg (sustained release) CAP
31. Venlafaxine 75mg (sustained release) CAP

Supplementary Table:

Table S1: The number of Qatari patients receiving prescriptions of antidepressants over a period of 2 years in (a) all the hospitals, and (b) Mental Health Hospital alone.

(a)

Age	< 20		20-29		30-39		40-49		50-59		>= 60		Total
Sex	Female	Male	Female	Male	Female	Male	Female	Male	Female	Male	Female	Male	
escitalopram	99	63	272	268	414	332	450	323	422	229	575	328	3775
amitriptyline	40	20	221	208	361	237	535	257	499	211	520	291	3400
duloxetine	7	2	56	69	187	126	319	165	508	242	770	365	2816
fluoxetine	35	29	115	130	153	110	173	97	148	59	124	68	1241
mirtazapine	13	5	41	66	73	79	91	82	106	71	305	187	1119
paroxetine	5	5	24	43	44	54	59	59	59	42	54	28	476
sertraline	18	13	39	45	44	49	49	28	27	21	42	30	405
venlafaxine	4	10	16	29	35	50	53	33	59	30	47	28	394
fluphenazine/nortriptyline	3	2	14	18	33	27	50	32	68	24	80	26	377
imipramine	8	5	13	12	27	18	29	15	28	12	17	21	205
clomipramine	0	1	7	13	5	28	8	30	7	29	12	19	159
fluvoxamine	2	7	10	10	11	14	9	14	14	12	21	7	131
amitriptyline/ chlordiazepoxide	1	1	3	4	2	3	7	5	5	4	7	2	44
trimipramine	0	0	0	2	1	3	3	0	3	3	8	3	26
maprotiline	0	0	0	0	1	0	1	0	7	3	9	3	24
selegiline	0	0	0	0	0	0	0	1	0	1	3	4	9
Total	235	163	831	917	1391	1130	1836	1141	1960	993	2594	1410	14601

(b)

Age	< 20		20-29		30-39		40-49		50-59		>= 60		Total
Sex	Female	Male	Female	Male	Female	Male	Female	Male	Female	Male	Female	Male	
escitalopram	39	19	65	84	95	102	94	71	75	55	118	68	885
mirtazapine	10	3	29	46	42	55	54	57	61	41	168	99	665
fluoxetine	24	16	53	62	55	53	57	40	43	20	38	10	471
sertraline	16	11	28	30	28	34	29	19	18	8	25	20	266
venlafaxine	3	9	8	18	14	35	27	24	40	23	30	15	246
paroxetine	3	4	12	13	25	30	32	22	30	16	26	8	221
duloxetine	2	0	3	10	20	22	17	15	23	10	36	16	174
amitriptyline	1	0	6	6	16	12	24	8	22	7	12	8	122
fluphenazine/nortriptyline	0	0	4	1	12	2	25	6	27	6	30	7	120
fluvoxamine	1	5	5	4	7	10	7	7	11	9	14	4	84
clomipramine	0	0	6	4	5	11	4	6	2	6	9	3	56
amitriptyline/ chlordiazepoxide	1	1	3	4	2	3	6	4	4	4	4	2	38
maprotiline	0	0	0	0	0	0	1	0	5	1	8	2	17
imipramine	0	0	0	2	2	0	2	0	1	1	2	5	15
trimipramine	0	0	0	0	0	0	2	0	1	3	6	3	15
Total	100	68	222	284	323	369	381	279	363	210	526	270	3395
